# Efficacy of Health Surveillance and Polymerase Chain Reaction Testing in Judo During the COVID-19 Pandemic

**DOI:** 10.7759/cureus.57898

**Published:** 2024-04-09

**Authors:** Naoki Sakuyama, Naohisa Fujita, Akira Ikumi, Masaomi Miura, Shinji Nagahiro, Mikami Yasuo

**Affiliations:** 1 Medical Science Committee, All Japan Judo Federation, Bunkyo, JPN; 2 Frontier Surgery, The Institute of Medical Science, The University of Tokyo, Minato-ku, JPN; 3 Infectious Diseases & Infection Prevention, Kyoto Prefectural Institute of Public Health and Environmental Sciences, Kyoto, JPN; 4 Orthopedic Surgery, The University of Tsukuba, Tsukuba, JPN; 5 Diabetes and Metabolic Diseases, The University of Tokyo Hospital, Tokyo, JPN; 6 Neurosurgery, Yoshinogawa Hospital, Tokushima, JPN; 7 Rehabilitation Medicine, Kyoto Prefectural University of Medicine, Kyoto, JPN

**Keywords:** health surveys, judo, pcr, pandemic, covid-19

## Abstract

Background

The COVID-19 pandemic necessitated infection control for all sporting activities. More careful infection control measures are required in judo, where close contact with opponents cannot be avoided. The Medical Science Committee of the All Japan Judo Federation (AJJF) established infection control guidelines for daily practice and competitions. Infection control measures were also implemented at the national tournament organized by the AJJF.

Objective and methods

This study aimed to examine the effectiveness of pre-tournament health surveys and PCR testing in guidelines for judo tournaments. Participants had to complete a health survey one to two weeks before the tournament. Initially, PCR testing was performed on all athletes; however, the final policy was to conduct PCR testing only on athletes with an infected person (risk team testing method). The effectiveness of these methods was also examined.

Results

In 16 competitions between October 2020 and March 2023, 6980 contestants were registered, and PCR testing was performed on 3672 athletes; 29 (0.79%) had a positive PCR test. Only two contestants were unable to attend the tournament because of the health survey. No competition-related cluster outbreaks were observed. From May 2022, the competition was held under the guideline that only teams at risk of infection were tested and could only compete when they tested negative. No teams were tested according to this guideline. In the competitions organized within this guideline, only one person could not compete because of the information provided in the health survey. No clusters were observed in any of the competitions. The incidence of COVID-19 infection in the first week after the convention was 20 (0.60%) in testing only at-risk teams and 21 (0.57%) in testing all competitors, which was not significantly different.(p=0.62)

Conclusion

During the COVID-19 epidemic, health surveillance was necessary to prevent the registration of competitors at risk of infection prior to tournaments. If teams at risk of infection could be identified, PCR testing of all athletes might not be mandatory, and competitions could be organized safely. The Judo infectious disease control guidelines we have developed might be used for other contact sports in the future when other infectious diseases are prevalent.

## Introduction

The first COVID-19 infection was reported in China [1], and in March 2024, approximately 705 million people worldwide had been infected and 7 million had died [2]. In Japan, 34 million people had been infected, and 75,000 had died [2]. In Japan, a state of emergency was declared in infection-endemic regions [3]. Subsequently, the status of COVID-19 and infection control measures were improved, and it was reclassified as an infectious disease similar to influenza in May 2023 [4].

The impact of this outbreak on sports has been significant. Many guidelines for infection control in sports have been developed such as those for the National Collegiate Athletics Association. As a result of these guidelines, sports have resumed [5,6].

National sports organizations have periodically developed infection control guidelines in response to the epidemic's high impact [7,8].

Judo is a sport involving a high level of close contact. The International Judo Federation (IJF) has published guidelines for COVID-19 [9]. The All Japan Judo Federation (AJJF) has considered infection control measures for judo that are specific to this close-contact sport. We prepared guidelines for judo competitions announced to dojos and instructors throughout Japan [10].

Infection control measures are also needed to conduct tournaments and enforce infection control measures during judo practice.

In organizing a tournament that brings together athletes from various regions, it was decided to eliminate athletes at risk of COVID-19 by conducting health surveys in advance and holding tournaments with PCR testing to control the spread of the disease. PCR tests were performed as necessary when organizing the tournament during the COVID-19 pandemic. The testing was conducted using saliva samples. The effectiveness of saliva testing has already been demonstrated, and it is safer than nasopharyngeal testing when performed by individual players [11].

The policy was then changed to testing only those teams at risk of infection. After the pandemic ended, tournaments were held without testing.

In the present study, we hypothesized that pre-competition health surveillance of judo during the COVID-19 pandemic and PCR testing of athletes at risk of infection would effectively reduce the risk of COVID-19 infection during competitions.

No study has examined the outcomes of tournaments that have implemented preventive measures. The current study examined the effectiveness of a health survey administered before judo competitions and PCR testing of athletes during the COVID-19 pandemic.

## Materials and methods

Health surveys

When the AJJF decided to hold tournaments, athletes and convention officials were asked to complete a health survey two weeks before the convention date. Eventually, the survey period was shortened to one week.

The COVID-19 health survey included symptoms of fever, coughing, sneezing, sore throat, taste disorder, olfactory disorder, fatigue, diarrhea, vomiting, abdominal pain, and dyspnea [12].

A body temperature ˂0.5°C above average was considered normal while a body temperature ≥0.5°C higher than the average was considered fever [13]. Athletes were asked to refrain from practice and other activities two weeks to five days before the event. PCR testing was performed one to seven days before the event, as some participants came in advance from rural areas to participate in the competition. Participants with negative PCR test results were isolated as far as possible to avoid the risk of infection. PCR testing was conducted using reverse transcription (RT)-PCR or loop-mediated isothermal amplification.

Testing procedures

Since May 2022, PCR testing has not been required if there is no risk of infection such as an infected team member or a person in close contact with an infected team member. Hereafter, this method is referred to as the risk team method.

On the day PCR testing was performed, those with abnormal health surveys were sent to the hospital for examination without a PCR test, and the individual was not allowed to participate in the tournament.

The health survey was deemed abnormal if the individual had symptoms for more than three consecutive days two weeks before the examination, two or more days from one week to four days before, or one day from three days before the examination.

This rule was also applied to admissions to the convention center, and those who were symptomatic were not allowed to enter, even if their PCR test was negative.

From December 2020 to April 2022, PCR testing was enforced on all registered players, and if a team was infected with COVID-19 within two weeks of the tournament, players from that team were not allowed to compete in the tournament. We defined these groups as the Normal test groups (NGs). From May 2022, if a team had an outbreak within one week of the tournament, PCR testing was administered to the team’s tournament registrants in advance. If the test results were negative, the team was allowed to participate in the tournament. We defined these groups as the Risk team test groups (RGs).

In the case of an affiliation where there was concentrated contact before the competition, the focused contact was not allowed to compete. The other affiliated athletes were permitted to compete with a negative PCR test.

The definition of “concentrated contact person” followed the Ministry of Health, Labour, and Welfare guidelines. A person in close contact was defined as follows: 1) a person living with or having a long period of contact (including being in the same car, aircraft, etc.) with a patient (confirmed case); 2) a person with a high likelihood of having had direct contact with respiratory secretions, bodily fluids, or other contaminants of a patient (confirmed case); or 3) a person in contact with a patient (confirmed case) within touching distance (roughly 1 m) for at least 15 min (transmissibility is assessed comprehensively based on factors such as the surrounding environment and the nature of contact) [3].

Procedures for positive tests

Individuals with a positive PCR test before the match were promptly diagnosed at the hospital, registered at the health center, and treated. Subjects whose tests were suspicious or impossible to determine were retested at a medical center and allowed to compete if they tested negative.

Outbreaks of COVID-19 were followed up for one week after the competitions to see if they had occurred.

Statistical analysis

The athletes involved in the tournament were categorized into various groups and examined using Pearson’s χ2 test. All calculated p-values were two-sided, and statistical significance was set at P<0.05. All statistical analyses were performed using JMP 13 software (SAS Institute, Cary, NC, USA).

## Results

**Figure 1:**
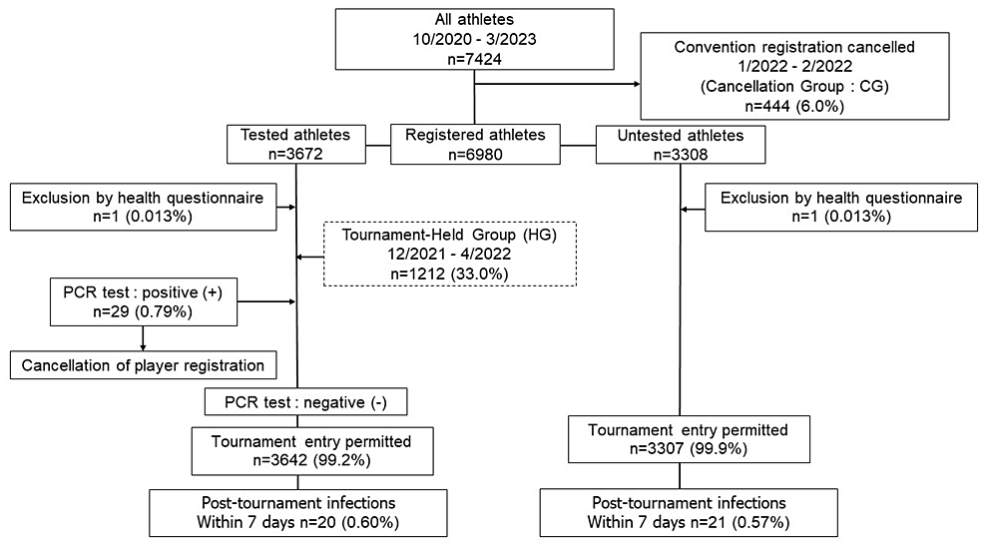
Flow chart of the study

Seventeen competitions were scheduled between October 2020 and March 2023, of which 16 competitions were held. From a total of 7424 athletes, 444 athletes who canceled one competition, hereafter defined as Cancellation Group (CG), were excluded. Finally, 6980 athletes participated (Figure 1).

The first two competitions provided for PCR testing to be conducted immediately prior to the competition while the other competitions provided for testing to be conducted immediately or by post. Health surveys were conducted at all competitions, and two athletes with abnormalities were deemed ineligible for testing.

PCR testing was performed on 3672 athletes (Table 1).

**Table 1:** Participants and background

Participants/Number of tournaments held	7424/17
Holding tournaments/Cancelled tournaments	16/1
Testing in the tournaments	11 (64.7%)
PCR testing	3672 (55.0%)
Male/Female	3872 (53.4%)/3552 (46.6%)
Age*	18 (15-35)

The PCR test results were positive in 29 individuals (5.3%). Forty-one (0.096%) infections occurred within a week of the convention. During the delta-variant outbreak, 444 athletes were scheduled to compete in one event. However, the event was canceled because 74 (16.7%) athletes were found to be infected or in close contact with infected persons and were withdrawn from the study. The incidence of infection within one week after the tournament was compared between the CG and the 1212 athletes who participated in a tournament held simultaneously, hereafter defined as the tournament-held group (HG). There were 10 (0.83 %) cases of infection in the week after the tournament in the HG and 4 (0.90 %) in the CG compared to the HG and the CG, which could hold the tournament, and there was no significant difference. (p=0.88) (Table 2).

**Table 2:** Results of the comparison of participants in the pandemic phase of COVID-19 *p< 0.05

n=1656/span	Tournament Held Group(HG) Dec/2021-April/2022	Cancellation Group (CG) Jan/2022-Feb/2022	p
Number of tests	1212	444	-
Positive tests	17 (1.4%)	-	-
Tournament participation refusal for the prior infection	5 (0.41%)	3 (0.68%)	0.49
Post-tournament infections within 7 days	10 (0.83%)	4 (0.90%)	0.88

The group of athletes considered for introducing RG comprised 3308 athletes in five competitions held between May 2022 and May 2023. The NG group from December 2020 to April 2022, in which the normal PCR test was performed, included 3672 participants and was compared to the RG from May 2022 to May 2023. Each group had one player who was not allowed to compete due to the results of their health survey.

The incidence of COVID-19 infection in the first week after the convention was 20 (0.60%) in RG and 21 (0.57%) in NG, which was not significantly different (p=0.62) (Table 3).

**Table 3:** Results of comparison between the Normal test group and the Risk team test group *p< 0.05

n=6980/span	Normal test group (NG) Dec/2020-April/2022	Risk team test group (RG) May/2022-March/2023	p
Number of tests	3672 (52.6%)	3308 (47.4%)	-
Positive tests	29 (0.79%)	-	-
Exclusion by health questionnaire	1 (0.013%)	1 (0.013%)	0.94
Post-tournament infections within 7 days	20 (0.60%)	21 (0.57%)	0.62

No clusters were reported at any competition.

## Discussion

Judo is a unique sport in which close contact is inevitable, such as during throws and ground holds, and infection control measures must be stricter than those in other sports. There are no reports that judo, a sport involving contact, does not spread infections in a strongly infectious disease.

We published the Judo Infection Control Guidelines from a medical perspective in April 2020, with seven revisions through July 2023 and the Convention’s Infection Control Measures [10]. The need for PCR testing was left to the discretion of the local convention organizers and was not necessarily mandatory [14,15]. We have already reported once on the stability of these guidelines and the effectiveness of PCR testing [13].

When the AJJF organizes tournaments, it attracts athletes from all over the country. It is difficult to monitor the infection situation across the country, and it is necessary to monitor the infection situation in athletes to develop guidelines.

In the tournaments from December 2020 to April 2022, 29 (0.79%) of the 3672 tests administered to athletes were positive. The tournament would have been held safely if symptomatic persons in the health surveys and pre-infected/concentrated contact individuals had been excluded.

Since December 2021, the incidence of wild-type, R1, and alpha variants has decreased, whereas the incidence of delta variants has increased [16]. In January 2022, there were 276 infections per 100,000 individuals [16]. The biggest warning in Japan occurred with the testing of 1212 athletes in that period, during which the virus was detected in 17 (1.4%) individuals, significantly more than the 12 (0.48%) among the 2460 contestants in the previous competition (p = 0.015).

However, in a follow-up survey one week after the tournament, which was canceled due to frequent match withdrawals during the same time, there was no difference in the number of cases considered to be associated with COVID-19 (4 (0.90%) vs. 10 (0.83%) at the time of the tournament; p = 0.88) (Table 2). There was also no significant difference in the number of participants who withdrew from registration due to infection before the competition (5 (0.41%) vs. 3 (0.68%); p = 0.49) (Table 2).

These results suggest that, first, the health surveys conducted before the games educated the participants about limiting the risk of infection. They also suggest that the health survey may have discouraged athletes at risk of infection from registering for the tournament.

We believe that the comparison of the number of infected players after the end of the tournament shows that the tournament can be safely organized if players at risk of infection are excluded in advance, even if PCR testing is not performed on all players participating in the tournament.

The limitation of this study is that no risk assessment was conducted on registered athletes. At the time, knowledge about COVID-19 was already widespread in Japan, so it is possible that some players or teams with the risk of infection did not participate in the competition. In addition, there were no tests done on support staff who directly practice with players associated with the team.

## Conclusions

During the COVID-19 epidemic, how competition management safely conducted pre-competition health surveillance and only tested athletes at risk of infection may have prevented competition-related infections. The guidelines we have developed for the safe operation of the contact sport of judo during an infectious disease outbreak might be used in other infectious disease outbreaks in the future.
